# Tantalum disulfide quantum dots: preparation, structure, and properties

**DOI:** 10.1186/s11671-020-3250-1

**Published:** 2020-01-28

**Authors:** Liangliang Zhou, Chuli Sun, Xueming Li, Libin Tang, Wei Guo, Lin Luo, Meng Zhang, Kar Seng Teng, Fuli Qian, Chaoyu Lu, Jing Liang, Yugui Yao, Shu Ping Lau

**Affiliations:** 1 Key Laboratory of Advanced Technique & Preparation for Renewable Energy Materials, Ministry of Education, Yunnan Normal University, Kunming, 650500 People’s Republic of China; 2 Kunming Institute of Physics, Kunming, 650223 People’s Republic of China; 3 School of Physics, Beijing Institute of Technology, Beijing, 100081 People’s Republic of China; 4 Institute of Environment and Health, Jianghan University, Wuhan, 430056 People’s Republic of China; 5 Teng College of Engineering, Swansea University, Bay Campus, Fabian Way, Swansea, SA1 8EN UK; 6 Department of Applied Physics, The Hong Kong Polytechnic University, Hong Kong, SAR People’s Republic of China

**Keywords:** Transition metal dichalcogenides, Quantum dots, Ultrasonic method, First-principle, Modulating bandgap

## Abstract

**Abstract:**

Tantalum disulfide (TaS_2_) two-dimensional film material has attracted wide attention due to its unique optical and electrical properties. In this work, we report the preparation of 1 T-TaS_2_ quantum dots (1 T-TaS_2_ QDs) by top-down method. Herein, we prepared the TaS_2_ QDs having a monodisperse grain size of around 3 nm by an effective ultrasonic liquid phase exfoliation method. Optical studies using UV-Vis, PL, and PLE techniques on the as-prepared TaS_2_ QDs exhibited ultraviolet absorption at 283 nm. Furthermore, we found that dimension reduction of TaS_2_ has led to a modification of the band gap, namely a transition from indirect to direct band gap, which is explained using first-principle calculations. By using quinine as reference, the fluorescence quantum yield is 45.6%. Therefore, our results suggest TaS_2_ QDs have unique and extraordinary optical properties. Moreover, the low-cost, facile method of producing high quality TaS_2_ QDs in this work is ideal for mass production to ensure commercial viability of devices based on this material.

**Graphical abstract:**

TaS_2_ quantum dots having a monodisperse grain size of around 3 nm have been prepared by an ultrasonic liquid phase exfoliation method, it has been found that the dimension reduction of TaS_2_ has led to a transition from indirect to direct band gap that results in the unique and extraordinary optical properties (PL QY: 45.6%). 
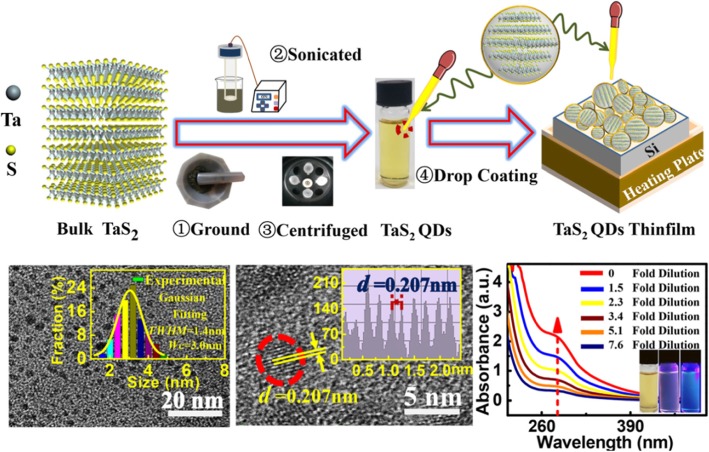

## Introduction

Recently, the family of layered transition metal dichalcogenides (LTMDs) [1] has drawn great attention in many fields, such as electronic devices [2], energy storage [3], catalysis [4], bio-imaging [5, 6], and biosensing [7], due to its many interesting physical and electrical properties. Typically, the structure of LTMDs is formed [1] by covalently bonded monolayer, and each monolayer is linked by Van der Waals forces; hence the LTMDs can be easily cleaved along the layer plane by either chemical or physical methods. According to previous work, the band gaps of LTMDs can be modified from indirect to direct band gap by decreasing the number of layers [8]. In particular, the TaS_2_ has been studied extensively for various applications, ranging from optical switch [9] to catalysis [10], as they exhibit tunable band gap, controllable size, and strong photoluminescence. Therefore, it is becoming a widely focused functional material.

At present, both top-down and bottom-up methods are adopted to prepare nanomaterials [11, 12]. The bottom-up approach is based on atoms and molecules as building blocks, which are used to form nanoparticle structures according to relevant purposes. This method mainly involves gas-phase and liquid-phase reactions [13, 14]. As for the top-down approach, electrochemical and etching methods [15, 16] have been applied to prepare TaS_2_ nanomaterials by weakening the Van der Waals interactions and cleaving the bonding force between the layers to obtain TaS_2_ nanomaterials from its bulk materials. For example, Zeng et al. [17] prepared TaS_2_ nanosheets through lithium interaction and exfoliation by controlling the cut-off voltage. Zhang et al. [18] prepared monodispersed TaS_2_ nanodots by a facile top-down method. QDs [19–22] has attracted wide interest worldwide in recent years, but TaS_2_ QDs are rarely reported. Therefore, facile methods are still in need to prepare industry applicable TaS_2_ QDs with narrow size distribution and good dispersion.

In this study, TaS_2_ QDs with a monodisperse grain size of around 3 nm were prepared by ultrasonic method. In this process, the Van der Waals interactions in between the TaS_2_ layers were first weakened by intercalation of NMP and then followed by further exfoliation using high power ultrasonic energy. The size of TaS_2_ QDs can be easily tuned by adjusting centrifugation rate and time; a higher centrifugation rate and a bigger centrifugation time result in a smaller size. This provides an efficient and practical method in the preparation of TaS_2_ QDs. Its structural, electronic, and optical properties were characterized by experiments, as well as first-principle calculations.

## Methods

The TaS_2_ powder was purchased from Aladdin Company (Chengdu China, purity ≥ 99.99%). The chemical reagents were purchased from Sinopharm Chemical Reagent Co. Ltd and used as received: N-methyl-2-pyrritolidone (NMP) (purity ≥ 99.0%) and ethanol (purity ≥ 99.7%).

### Preparation of TaS_2_ QDs

TaS_2_ powder 0.5 g was grinded in the mortar for 2 h. Fifty milliliters of NMP solvent was added to the grinded powder sample. The mixture was then ultrasonic treated for 4 h with an ultrasonic power of 210 W. The suspension after ultrasonic treatment was centrifuged at the rate of 7000 rpm for 25 min. The supernatant, which obtains the TaS_2_ QDs, was collected.

### General Characterization

The morphology, elemental composition, and size distribution of TaS_2_ QDs were studied using transmission electron microscopy (TEM, Tecnai G2 TF30 S-Twin), atomic force microscopy (AFM, Seiko SPA-400), scanning electron microscopy (SEM, SUPRA 55VP), and energy-dispersive spectroscopy (EDS, X-Max20). TaS_2_ QDs suspension was drop-casted onto an ultrathin carbon-coated holey support film, consisting of 300 mesh copper grids, during TEM characterization. The phase structure of TaS_2_ QDs was characterized by X-ray photoelectron microscopy (XPS, PHI Versa probe II), X-ray diffractometer (XRD, UItima IV, X-ray source: Cu Kα, *λ* = 1.54178 Å), Fourier-transform infrared spectrometer (FTIR, Nicolet iS10) using the KBr pellet technique, and Raman spectroscopy (Renishaw in Via) using an argon-ion laser having an excitation wavelength of 514.5 nm. The optical properties of TaS_2_ QDs were characterized using UV-visible spectrophotometer (UV-Vis, Shimadzu UV-3600) and fluorescence photoluminescence spectrometer (PL and PLE, Hitachi, F-4500).

## Results and Discussion

**Fig. 1:**
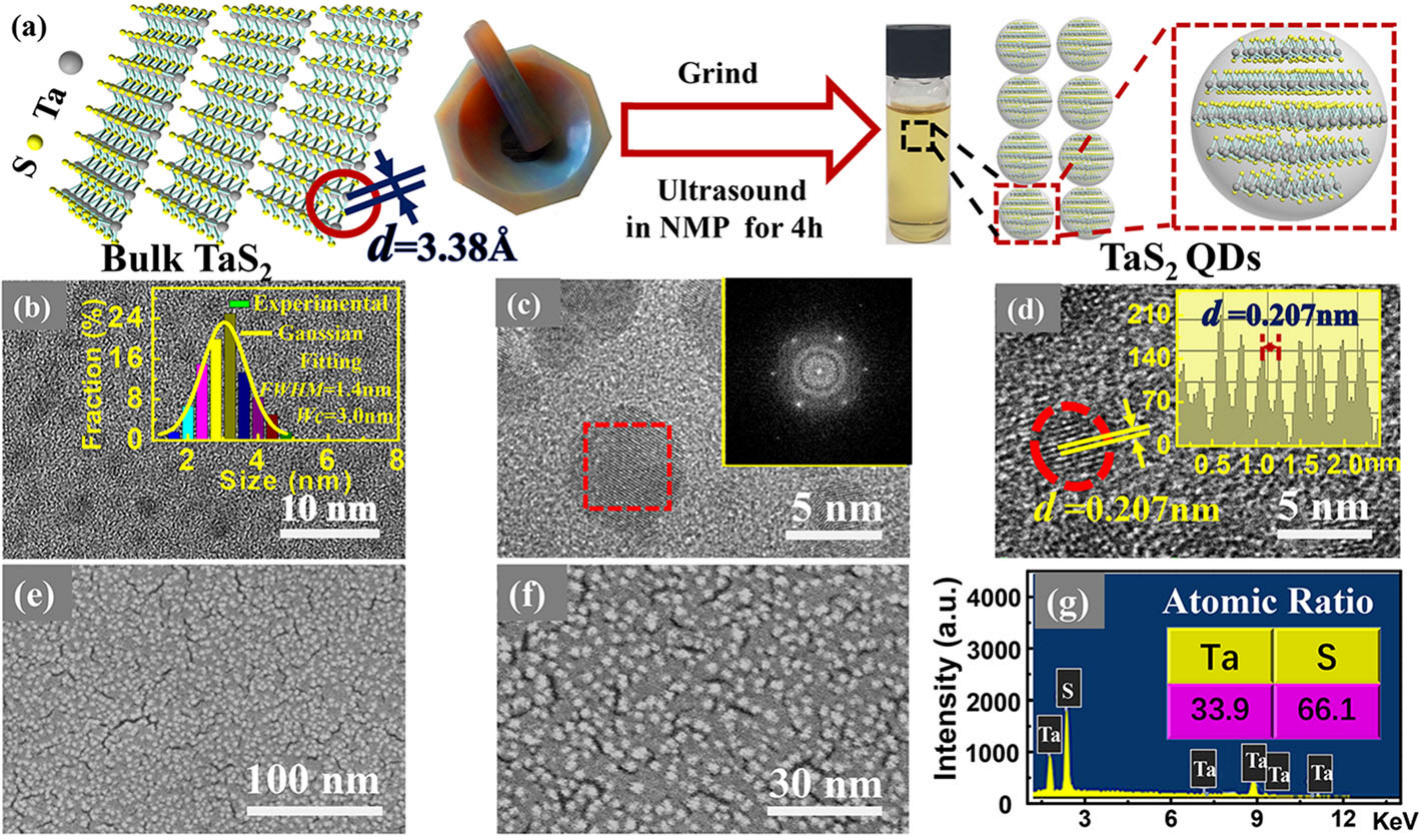
**a** Schematic diagram showing the process of TaS_2_ QDs formation; **b** TEM image of the TaS_2_ QDs, inset shows TaS_2_ QDs particle size distribution. Gaussian fitting curve is shown as yellow line; **c** FFT pattern (inset) of a selected area (dotted red square); **d** HR-TEM image of the TaS_2_ QDs, inset shows the line profile of the diffraction fringes; **e** SEM image at 70.0 K; **f** SEM image at 100.0 K; **g** EDS spectrum of TaS_2_ QDs

The process of TaS_2_ QDs formation from its bulk crystal is depicted in Fig. 1a. The preparation process consisted of three steps, namely grinding, ultrasonic, and centrifugation. An enlarged schematic of TaS_2_ QDs is shown within the dotted red square of Fig. 1a. The tawny solution in the sample bottle was TaS_2_ QDs solution after centrifugation. Figure 1 b shows the TEM image of the TaS_2_ QDs, which are spherical in shape with uniform size distribution. As shown in the inset, the size distribution of the TaS_2_ QDs followed a Gaussian fitted curve with an average diameter of *W*_*C*_ = 3.0 nm and full width at half maximum (*FWHM*) of 1.4 nm. It was reported that the thickness of the TaS_2_ monolayer ranged from 0.6 to 1.2 nm [23]; hence, the QDs could comprise of 2–5 layers of TaS_2_. The number of TaS_2_ layer can be reduced by increasing the centrifugal rate and time (as shown in Additional file 1: Fig. S1).

The FFT pattern is shown in the inset of Fig. 1c. It shows a hexagonal crystalline structure, which corresponds to the TaS_2_ QDs structure in Fig. 1a. As shown in Fig. 1d, the TaS_2_ QDs line profile exhibits obvious lattice stripe with a spacing of 0.207 nm. The SEM image in Fig. 1e shows a uniform distribution of TaS_2_ QDs, thus indicating a good dispersibility and uniform size distribution. At higher SEM magnification, it is apparent that the surface consisted of rugged particles as shown in Fig. 1f. This indicates the formation of independent spherical TaS_2_ QDs during the preparation process. EDS technique was used to characterize the elemental composition as shown in Fig. 1g. A film of TaS_2_ QD_S_ was deposited on copper sheet during the EDS characterization in order to avoid the overlapping of Ta and Si/SiO_2_ substrate peaks, which could complicate the analysis. The measured atomic percentage ratio of Ta and S in the material was 33.9/66.1 ≈ 1:1.95, which is close to the theoretical value of 1:2.

**Fig. 2:**
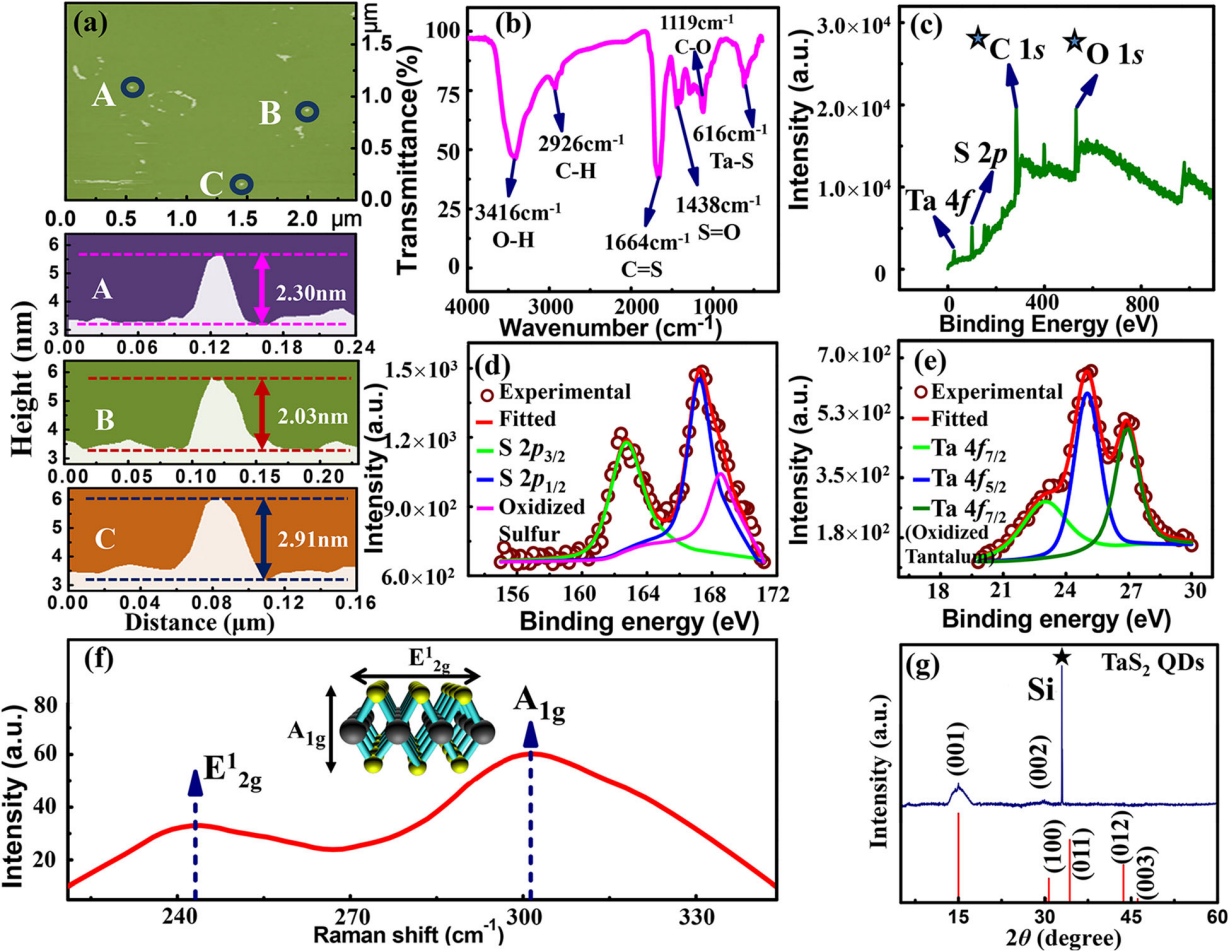
**a** AFM morphology and height analysis results of TaS_2_ QDs, labeled A, B and C, were randomly selected three points; **b** TaS_2_ QDs FTIR spectrum; **c** the full-scan XPS spectrum of the TaS_2_ QDs; **d** XPS spectrum of S 2*p*; **e** XPS spectrum of Ta 4*f*; **f** The Raman vibration mode of TaS_2_ QDs and Raman spectra of TaS_2_ QDs; **g** XRD diffraction pattern of TaS_2_ QDs

**Fig. 3:**
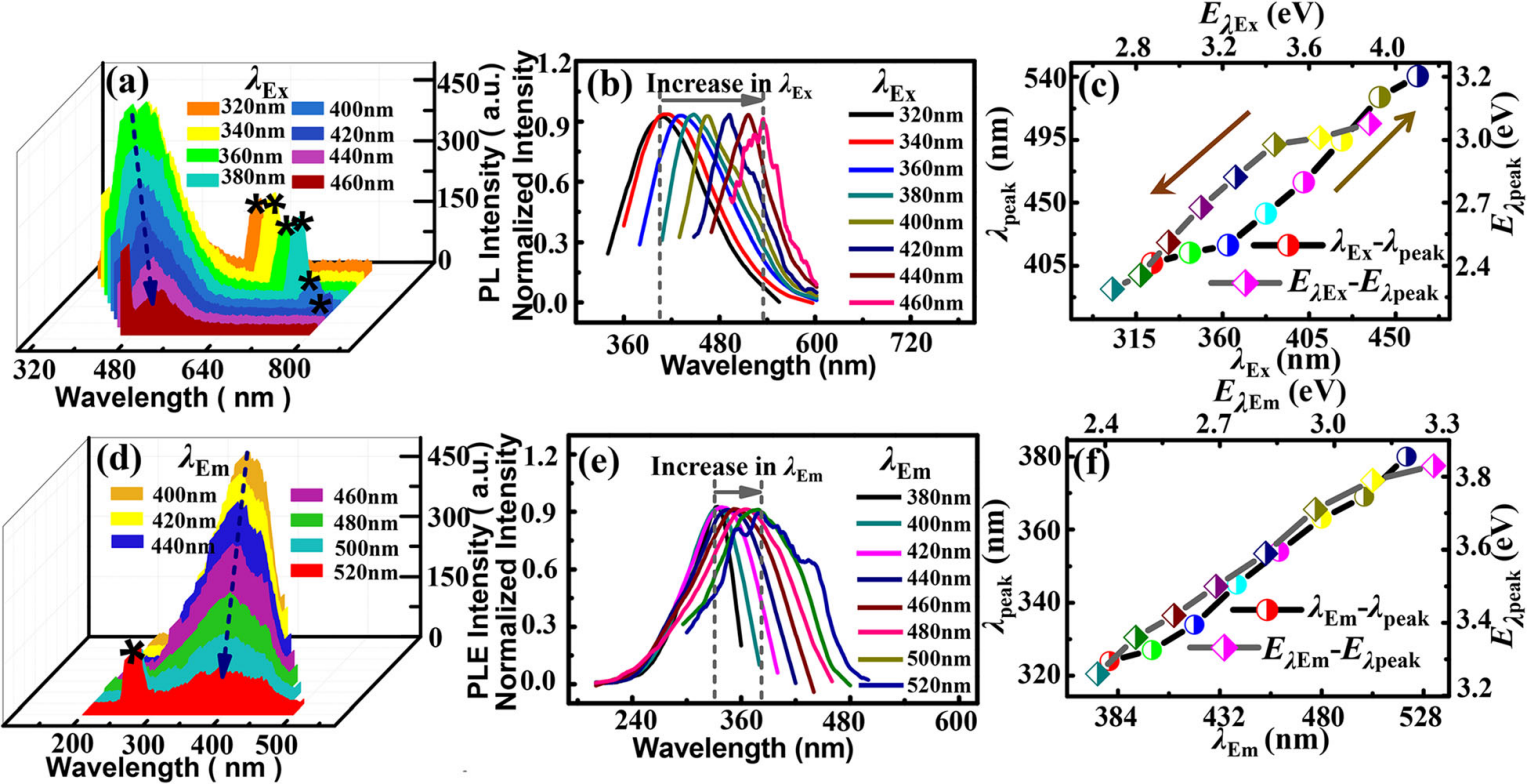
**a**, **d** The PL & PLE spectra of TaS_2_ QDs [* interference peak (*λ*_Ex_ and *λ*_Em_) from the instrument], respectively; **b**, **e** The PL and PLE normalized spectra of TaS_2_ QDs under different *λ*_Ex_ and *λ*_Em_, respectively; **c**, **f** the relationship of peak and energy for TaS_2_ QDs

Figure 2 a shows AFM images of the TaS_2_ QDs, labeled as A, B, and C, which were randomly selected and their heights were measured to be 2.30 nm, 2.03 nm, and 2.91 nm, respectively. The average height of 2.41 nm is consistent with the average diameter of 3.01 nm measured using TEM. The FTIR spectra, shown in Fig. 2b, reveal that the Ta-S bond stretching vibration absorption peak was situated at 616 cm^−1^. Figure 2 c shows Ta 4*f*, S 2*p*, ^★^C 1*s*, and ^★^O 1*s* peaks from XPS full-scan spectrum. The ^★^C 1*s* and ^★^O 1*s* peaks were impurity peaks produced by solvent NMP and oxide. Figure 2 d shows the XPS spectrum of S 2*p* deconvoluted into three components, namely S 2*p*_3/2_ (163.4 eV), S 2*p*_1/2_ (166.7 eV), and oxidized sulfur (168.2 eV). The XPS spectrum of Ta 4*f* is shown in Fig. 2e and was deconvoluted into components, such as Ta 4*f*_7/2_ (23.2 eV), Ta 4*f*_5/2_ (25.6 eV), and Ta 4*f*_7/2_ (27.2 eV). The Ta 4*f*_7/2_ peak at 27.2 eV is associated with oxidized tantalum [24, 25]; oxidation has also been observed in other QDs [26–28]. Figure 2 f shows the Raman vibration mode of TaS_2_ QDs. The E^1^_2g_ and A_1g_ modes relate to the in-plane and out-of-plane vibration respectively [29]. The A_1g_ and E^1^_2g_ modes of the TaS_2_ QDs were observed at 301.4 cm^−1^ and 242.3 cm^−1^ respectively. The Raman intensity of the E^1^_2g_ vibration mode was much smaller than that of A_1g_, which could due to the fact that A_1g_ mode is more sensitive to strain than the E^1^_2g_ mode in TaS_2_ QDs. It shows that the A_1g_ mode dominated during the preparation process of TaS_2_ QDs. Figure 2 g shows XRD diffraction pattern of the TaS_2_ QDs. When compared with space group P$\overline{3}$m1(164), the pattern indicates trigonal structure of 1 T-TaS_2_ [30]. According to the standard PDF#04-001-0068 card, the diffraction peak 2*θ* at 15.0° represented crystal plane (001) with *d* = 0.590 nm, which corresponds to the *C*-axis crystal surface spacing. The peak at 30.2° represented crystal plane (002) with *d* = 0.295 nm. The peak at 33.0° (asterisk peak) was originated from the Si (001) substrate [31]. The grain size can be calculated using the Debye-Scherrer (Eq. (1)) [32].

1 $$ D=\frac{0.89\lambda }{\beta \cos \theta } $$

where *D* is grain size, *β* is *FWHM* of the diffraction peak of the measured sample, *θ* is diffraction angle, and *λ* is X-ray wavelength. The calculated grain size of 3.8 nm using the strongest diffraction peak (001) is close to the TEM result of 3.01 nm. Figure 3 a and d show the PL and PLE spectra of the TaS_2_ QDs respectively, with excitation wavelength (*λ*_Ex_) varied from 320 nm to 460 nm and the emission wavelength (*λ*_Em_) changed from 400 to 520 nm at 20 nm step. The PL and PLE peaks were red-shifted, as indicated by the dark blue lines in Fig. 3a and d respectively. As shown in Fig. 3b and e, the red-shift of the normalized intensity peaks is more noticeable for the PL (e.g., from 391 to 519 nm) than the PLE (e.g., from 324 to 380 nm) spectra. The wavelength and energy-dependent PL and PLE peaks are shown in greater details in Fig. 3c and f respectively. The peak energies of the red-shifted excitation wavelength ranged from 3.17 to 2.39 eV, while the red-shifted emission energies ranged from 3.83 to 3.26 eV. It can be seen that a higher excitation energy (*λ*_Ex_ = 320 nm) led to a larger Stokes shift (71 nm), whereas a lower excitation energy (*λ*_Ex_ = 460 nm) resulted in a smaller Stokes shift (59 nm). The difference in Stokes shift is probably due to the size distribution of the prepared QDs, which has also been observed from Se QDs and Te QDs [27, 28]. Comparing the PL and PLE peaks, the PL peaks exhibited a greater red-shift than the PLE peaks, and with the increase of the peak wavelengths, the PL peak has a larger Stokes frequency shift than the PLE peak [33]. The red-shifted intensity peak indicates that the optical properties of TaS_2_ QDs have an obvious dependence on the wavelength.

**Fig. 4:**
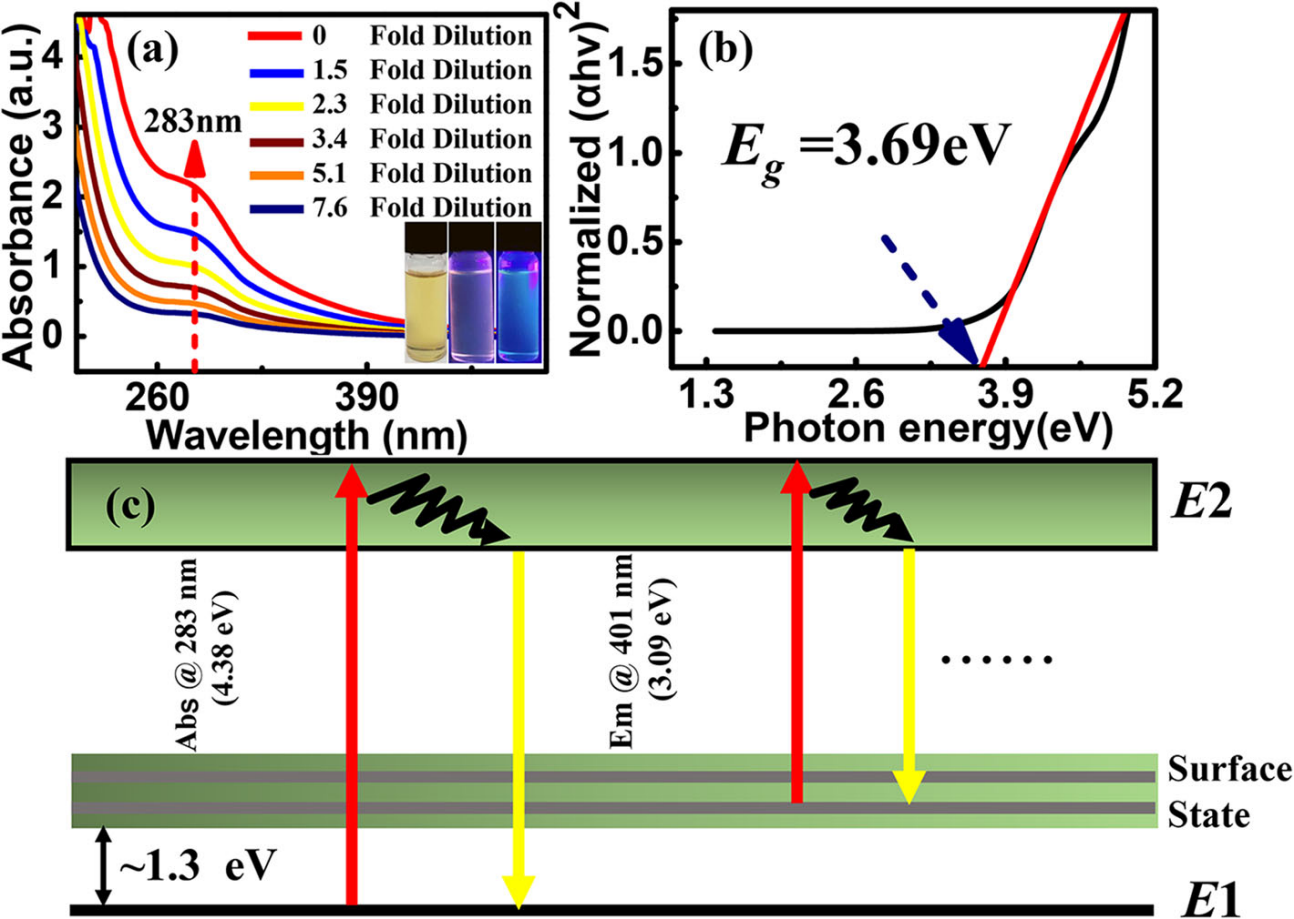
**a** UV-Vis absorption spectra of TaS_2_ QDs and TaS_2_ QDs in natural light, 254 nm and 365 nm UV light illumination; **b** the direct band gap spectrum of the TaS_2_ QDs by Tauc method; **c** energy level diagram of TaS_2_ QDs

**Fig. 5:**
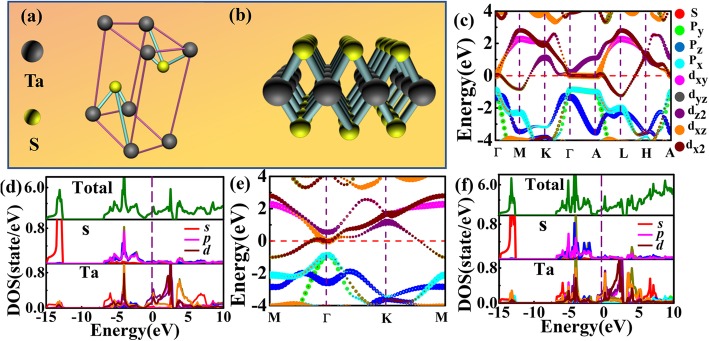
Structure of TaS_2_, **a** bulk TaS_2_ and **b** monolayer TaS_2_. **c**–**f** Band structure calculations by PBE functional. Partial band structures of bulk and monolayer TaS_2_ in **c** and **e**, respectively. Partial density of states (PDOS) of bulk and monolayer TaS_2_ in **d** and **f**, respectively

Figure 4a shows the UV-Vis absorption spectra of TaS_2_ QDs. An absorption peak was observed at 283 nm, which is caused by electron transition upon UV illumination. Based on the study of the reduction in the number of layers, a blue-shifted absorption peak was observed (as shown in Additional file 1: Fig. S2). In addition, the TaS_2_ QDs solution appeared yellow in color under natural light, peony in color under ultraviolet at 254 nm, and blue in color at 365 nm. Tauc mapping method was used to calculate the TaS_2_ QDs’ band gap spectrum, according to Eq. (2) [17, 34]:

2 $$ \alpha \mathrm{hv}=A{\left(\mathrm{hv}-{E}_g\right)}^{1/2} $$

where *α* is absorption coefficient, *A* is a constant, hv is light energy, and *E*_g_ is band gap energy. The TaS_2_ QDs has a direct band gap (*E*_g_ = 3.69 eV) as shown in Fig. 4b. The results indicate a reduction in the number of layers in TaS_2_ QDs would lead to a modification of the band gap, including indirect to direct band gap transitions. Based on the above results, TaS_2_ QDs energy level structure is proposed as shown in Fig. 4c. During electron transition (*E*1-*E*2), the energy level of TaS_2_ QDs is *E*_g_ = 4.38 eV. Due to the presence of surface energy level, an emission wavelength of 401 nm is observed and this corresponds to *E*_g_ = 3.09 eV. Therefore, the transition energy from *E*1 to surface state is about 1.3 eV. In order to study the influence of fluorescence effect caused by the increase in band gap, the fluorescence quantum yield (*Q*_*s*_) of TaS_2_ QDs was calculated using quinine (*Q*_*r*_= 0.54 in 24.9% ethanol) as reference, based on the following equation (Eq. 3) [35, 36]:

3 $$ {Q}_s={Q}_r\times \frac{I_s}{I_r}\times \frac{A_r}{A_s}\times {\left(\frac{n_s}{n_r}\right)}^2 $$

where the subscript _*s*_ denotes sample and _*r*_ indicates reference. _*Q*_ is PL quantum yield, _*I*_ is emitting peak area of fluorescence, *A* is absorbance at a specific excitation wavelength, and *n* is refractive index. The calculated fluorescenceyield of 45.6% indicates excellent fluorescence properties of the TaS_2_ QDs. First-principle calculations were performed to further investigate the reasons for the increase in band gap of TaS_2_ QDs. Figure 5 a and b show the bulk and monolayer structures of TaS_2_. For the monolayer TaS_2_, a vacuum of 29.5 Å in the *Z* direction was added when constructing the unit-cell. The calculations were performed using density functional theory (DFT) as implemented in the Vienna Ab initio simulation package (VASP) [37–39]. The electronic exchange-correlation effects were treated using the generalized gradient approximation (GGA) in the Perdew-Burke-Ernzerhof (PBE) form [40, 41]. When using Heyd-Scuseria-Ernzerhof (HSE) hybrid functionals [42, 43], 25% Hartree-Fock and 75% PBE-GGA were chosen for short-ranged exchange part in the HSE06 hybrid functionals. The projector augmented wave (PAW) method was utilized to treat the interactions between the ionic cores and the valence electrons [44, 45], where valence electron configuration of S and Ta were set as 3*S*^2^3*p*^4^ and 5*d*^3^6*s*^2^, respectively. The energy cut-off of plane wave basis was set to 520 eV. The Monkhorst-Pack grid mesh [46] of 11 × 11 × 7 and 11 × 11 × 1 were used to sample the Brillouin zone of the bulk and monolayer TaS_2_, respectively. The convergence in energy was set to 1 × 10^−5^ eV during electronic structure calculations. The electronic structures of bulk and monolayer TaS_2_ were calculated by PBE functional, as shown in Fig. 5c and e, respectively.

**Fig. 6:**
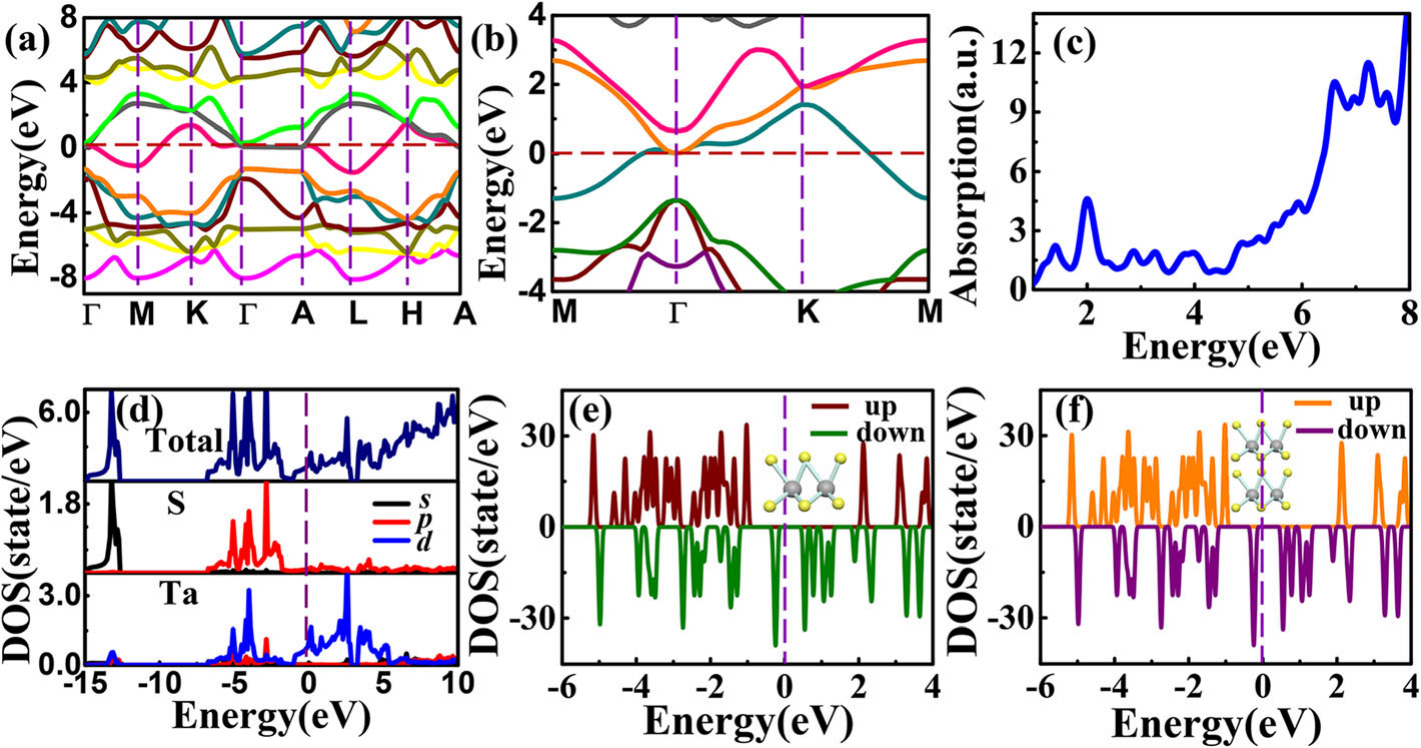
**a** Bulk and **b** monolayer TaS_2_ by HSE functionals. **c** Optical absorption spectra of monolayer TaS_2_. **d** PDOS by calculated by HSE. **e** The monolayer and **f** the two-layer DOS of QDs calculated by HSE

The results are in good agreement with previous calculations [47, 48]. Both the bulk and monolayer TaS_2_ have metallic in-phase states, and the band across the Fermi level is mostly composed of *d*_x2_ orbital of Ta atoms. The valence band is mainly composed of *p* orbitals of S atoms, while the conduction band is mainly from *d* orbitals of Ta atoms. At Γ point, an indirect gap is transiting to a direct gap from bulk to monolayer structure due to lacking of inter-plane interactions. The band structure was checked using HSE06 hybrid functionals (Fig. 6a, b). The results are similar to PBE except for a larger gap near Γ point for the HSE results, where the conduction band shifted toward lower energy for about 0.5 eV. The absorption spectrum of monolayer TaS_2_ was also calculated and it contains mainly four peaks at 1.41 eV, 2.00 eV, 6.61 eV, and 7.23 eV. Comparing the absorption spectra and the PDOS, as shown in Fig. 6c and d, the two peaks in the 0~2 eV region are provided by the S 3*p*→Ta 5*d* electronic transitions, and the S 3*p* → Ta 6 *s* electronic transitions contribute to the peaks in the 6~8 eV region.

Next, the TaS_2_ QDs were modeled as clusters with one and two-layer of Ta-2S unit and compared their spin-polarized DOS. As shown in Fig. 6e and f, due to the dangling bonds of S atoms at the surface, the spin-polarized DOS of the one- and two-layer models show half-metallic nature, where there is a ~ 3 eV gap for the spin-up electrons that is twice the gap at Γ point of the infinite 2D monolayer in Fig. 6b. This demonstrates strong quantum confinement effect. The gap of spin-up electrons of the one-layer model is slightly larger than the two-layer model as a result of lacking inter-plane interactions.

## Conclusions

TaS_2_ QDs having an average size of about 3 nm were prepared by ultrasonic method. The morphology and structural studies performed on the nanomaterials show that they have controllable and hexagonal honeycomb shape. The optical properties of the TaS_2_ QDs, including absorption and PL, were investigated. A red-shifted effect, compared to the bulk material, was observed and the QDs exhibited multicolor luminescence with strong absorption in near ultraviolet region. The band gap of the TaS_2_ QDs increased to 3.69 eV from indirect to direct band gap, hence exhibiting extraordinary optical properties. The indirect to direct transition and quantum confinement effect in the electronic structures were confirmed by first-principle calculations of a simple model of the QDs. These results will extend the application of TaS_2_ QDs in devices, such as photodetectors. Furthermore, the preparation method is also applicable to other layered materials to produce low-cost high-quality QDs from bulk materials.

## Supplementary information

**Additional file 1: Fig. S1. (a)** and **(c)** show the HR-TEM images of the TaS_2_ QDs after centrifugation at 16000 rpm for 30 minutes. **(b)** Particle size distribution of TaS_2_ QDs. **(d)** The line profile of the TaS_2_ QDs diffraction fringes. **Fig. S2. (a)** UV-Vis absorption spectra of TaS_2_ QDs after centrifugation at 16000 rpm for 30 minutes. **(b)** UV-Vis absorption spectra of TaS_2_ QDs compared between 16000 rpm for 30 minutes and 7000 rpm for 25 minutes. **(c)** and **(d)** show normalized PL spectra of TaS_2_ QDs with excitation wavelength (*λ*_Ex_) of 250 nm and 270 nm, respectively.

## Data Availability

The data supporting the conclusions of this article are included within the article and its additional files.

## Abbreviations

AFM: Atomic force microscopy
EDS: Energy-dispersive spectroscopy
FTIR: Fourier-transform infrared spectrometer
LTMDs: Layered transition metal dichalcogenides
NMP: N-methyl-2-pyrritolidone
PL and PLE: Fluorescence photoluminescence spectrometer
SEM: Scanning electron microscopy
TaS_2_: Tantalum disulfide
TEM: Transmission electron microscopy
UV-Vis: UV-visible spectrophotometer
XPS: X-ray photoelectron microscopy
XRD: X-ray diffraction
