# Supplementary material for: Tantalum disulfide quantum dots: preparation, structure, and properties
Source: Nanoscale Res Lett. 2020 Jan 28;15:20. doi: 10.1186/s11671-020-3250-1 (PMC6987292; doi:10.1186/s11671-020-3250-1)
Supplement: Supplementary file 1 — Additional file 1: Fig. S1. (a) and (c) show the HR-TEM images of the TaS2 QDs after centrifugation at 16000 rpm for 30 minutes. (b) Particle size distribution of TaS2 QDs. (d) The line profile of the TaS2 QDs diffraction fringes. Fig. S2. (a) UV-Vis absorption spectra of TaS2 QDs after centrifugation at 16000 rpm for 30 minutes. (b) UV-Vis absorption spectra of TaS2 QDs compared between 16000 rpm for 30 minutes and 7000 rpm for 25 minutes. (c) and (d) show normalized PL spectra of TaS2 QDs with excitation wavelength (λEx) of 250 nm and 270 nm, respectively. [file 11671_2020_3250_MOESM1_ESM.docx]

**Supplementary Information**

**Tantalum disulfide quantum dots: preparation, structure and properties**

Liangliang Zhou,^1^ Chuli Sun,^3^ Xueming Li,^1,*^ Libin Tang,^2,*^ Wei Guo,^3,*^ Lin Luo,^2^ Meng Zhang,^4^ Kar Seng Teng,^5^ Fuli Qian,^1^ Chaoyu Lu,^1^ Jing Liang,^1^ Yugui Yao,^3^ and Shu Ping Lau^6^

Fig. S1 shows the HR-TEM image of TaS_2_ QDs after centrifugation at 16000 rpm for 30 minutes. HR-TEM image and Gauss fitting of the particle size of TaS_2_ QDs are as shown in Fig. S1(a) and (b) respectively. From the figure, it can be seen that the average particle size of TaS_2_ QDs is 2.54 nm, which is corresponding to 2-4 layers, and the number of layers is less than that centrifuged at 7000 rpm for 25 minutes. Fig. S1(c) and (d) show the HR-TEM image of the lattice fringes of TaS_2_ QDs and the line profile of the diffraction fringes, respectively. The lattice spacing is 1.5 Å, which is smaller than the lattice spacing at 7000 rpm for 25 minutes, and the QDs show good dispersibility.

Fig. S2(a) shows the UV-Vis spectra of TaS_2_ QDs after centrifugation at 16000 rpm for 30 minutes, which reveals a strong absorption peak at 277 nm. A strong blue-shifted effect can be observed when compared to the absorption peak of TaS_2_ QDs centrifuged at 7000 rpm for 25 minutes as shown in Fig. S2(b). This may be related to the reduction in the number of layers. Fig. S2(c) and (d) show the PL spectra of TaS_2_ QDs at excitation wavelengths of 250 nm and 270 nm, respectively. Again, a significant blue-shifted effect can be observed when compared to spectra from 7000 rpm for 25 minutes. A blue-shift of 20 nm and 13 nm is recorded at excitation wavelength of 250 nm and 270 nm respectively, hence indicating a negative correlation between the modulation of the band gap and the number of layers.

| 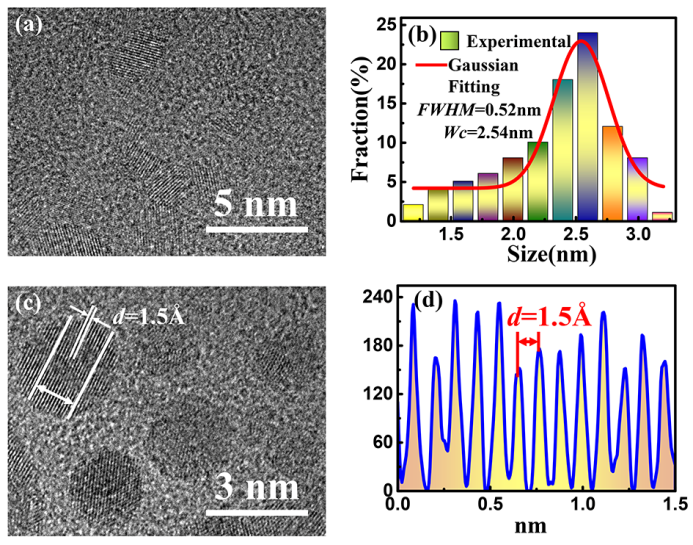  **Fig. S1. (a)** and **(c)** show the HR-TEM images of the TaS_2_ QDs after centrifugation at 16000 rpm for 30 minutes. **(b)** Particle size distribution of TaS_2_ QDs. **(d)** The line profile of the TaS_2_ QDs diffraction fringes. |
| --- |

| 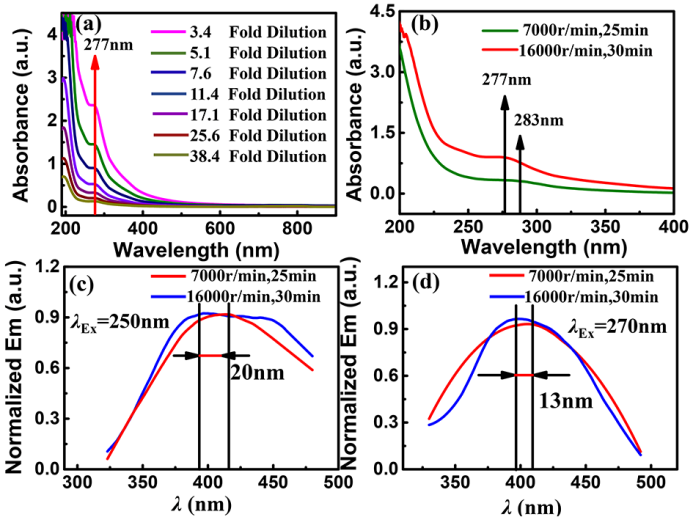  **Fig. S2. (a)** UV-Vis absorption spectra of TaS_2_ QDs after centrifugation at 16000 rpm for 30 minutes. **(b)** UV-Vis absorption spectra of TaS_2_ QDs compared between 16000 rpm for 30 minutes and 7000 rpm for 25 minutes. **(c)** and **(d)** show normalized PL spectra of TaS_2_ QDs with excitation wavelength (*λ*_Ex_) of 250 nm and 270 nm, respectively. |
| --- |
